# Behçet’s Disease and the Burden of Chronicity: Interplay Between Physical Symptoms, Mental Health, and Quality of Life

**DOI:** 10.7759/cureus.84660

**Published:** 2025-05-23

**Authors:** Mahmoud M Ramadan, Razan A Abdalla, Rama K Qoran, Rose A Daoud, Ghalya F Al-Nakawah, Ragad A Al-Khatib, Laila H Alheet, Abdulrahman E Alayyaf, Ousama Mahdi, Maamoun S Al-Hariri

**Affiliations:** 1 Cardiology, Faculty of Medicine, Mansoura University, Mansoura, EGY; 2 Clinical Sciences, College of Medicine, University of Sharjah, Sharjah, ARE; 3 Cardiology, University Hospital of Sharjah, Sharjah, ARE; 4 Ophthalmology, University Hospital of Sharjah, Sharjah, ARE

**Keywords:** anxiety, autoimmune, behçet, depression, multisystem, ocular manifestations, quality of life, relapsing-remitting, ulcers, vasculitis

## Abstract

Behçet's disease (BD) is a rare, chronic, multisystem inflammatory disorder that profoundly affects patients' quality of life (QoL) through complex physical, psychological, and social burdens. Characterized by recurrent oral and genital ulcers, ocular involvement, vascular inflammation, and systemic manifestations, BD leads to fluctuating yet progressive impairment across multiple domains.

Patients frequently experience significant declines in physical functioning, emotional well-being, and social participation, with higher rates of anxiety, depression, and cognitive disturbances than the general population. Studies report that over 50% of BD patients suffer from depression, and up to 60% experience anxiety disorders, with approximately 20% requiring psychotropic medications. Socioeconomic consequences, including unemployment, financial hardship, and social isolation, further exacerbate the disease burden.

Health-related QoL assessments highlight substantial variability influenced by disease severity, age, sex, and cultural context. Generic QoL instruments, such as the Short Form-36 Health Survey (SF-36), offer valuable comparisons across diseases, but disease-specific tools like the Behçet's Disease Quality of Life (BDQoL) scale provide superior sensitivity to BD-specific impairments. Comprehensive management strategies that combine immunosuppressive therapy, psychological interventions, and social support are essential to address the full impact of BD. Routine monitoring of QoL alongside clinical markers can guide more personalized, patient-centered care and improve long-term outcomes.

Early psychological screening and multidisciplinary interventions are particularly crucial in mitigating the bidirectional effects of physical and mental health deterioration. This review underscores the importance of adopting holistic, integrated approaches to BD management, prioritizing not only disease control but also the broader dimensions of patient well-being. Future research should focus on standardizing QoL measurements and developing longitudinal interventions tailored to the challenges of BD.

## Introduction and background

Behçet’s disease (BD) is a distinct clinical entity, characterized by oral aphthosis, genital ulceration, and iridocyclitis with hypopyon [1], being a systemic vasculitis of unknown etiology, predominantly affecting small and medium-caliber vessels, particularly the veins [2]. Diagnostic criteria require recurrent oral ulceration accompanied by any two of the following: recurrent genital ulceration, eye lesions, skin lesions, and a positive pathergy test (a skin hypersensitivity reaction to minor trauma) [3]. Although oral ulceration is typically essential for diagnosis, patients with systemic features characteristic of BD may still be diagnosed in its absence [4]. Management remains largely empirical, reflecting gaps in understanding of disease pathogenesis and therapeutic targets [5,6].

Quality of life (QoL) has emerged as a crucial outcome measure in the evaluation of many chronic disorders [7,8]. Given BD’s multisystem involvement, its impact on patients' daily lives and overall QoL is substantial. However, the extent to which these debilitating symptoms affect QoL in BD patients has not been fully elucidated [9].

This review highlights the multidimensional burden of BD on QoL, which is of increasing relevance to clinical practice. While definitive global trends remain difficult to quantify, some regional data suggest a potential rise in BD prevalence, particularly in countries along the historical Silk Road [10]. Given the complexity of the disease and its diverse systemic manifestations, this review explores the clinical, psychological, and socioeconomic dimensions of QoL in BD, outlines relevant assessment strategies, and discusses comprehensive, patient-centered management approaches aimed at improving long-term outcomes. The primary objective of this review is to synthesize current evidence on how BD impacts patients' QoL across clinical, psychological, and socioeconomic domains. By integrating findings from diverse studies, this review aims to inform clinicians, researchers, and policymakers about the unmet needs in BD care and highlight strategies that promote holistic and patient-centered management.

This review is organized into several sections: first, it summarizes the clinical features and pathophysiology of BD; next, it reviews the epidemiology and disease course. Subsequent sections discuss the conceptual framework and tools for assessing QoL, followed by an in-depth analysis of the physical, psychological, and socioeconomic factors influencing QoL. The final sections explore management strategies and implications for clinical practice.

## Review

Clinical presentation

BD typically manifests with recurrent oral and genital ulcers alongside systemic involvement [2]. This can sometimes elicit hoarseness and aphthous pharyngitis, and a subtle systemic inflammatory response [11,12]. Clinicians need to consider this organic cause when presented with a chronic complaint of sore throat and hoarseness in young adults. BD often exhibits a significant delay between initial symptoms and formal diagnosis. Recent data underscore that this latency can span several years in many regions. For example, a population-based study in Norway found a median diagnostic delay of about five years (symptom onset at mean age 24 vs. diagnosis at 33) [13]. Similarly, a registry in Wales (UK) reported a median diagnosis age of 42, notably older than in other cohorts - an outcome attributed to diagnostic delays (primary care providers’ unfamiliarity with BD and severe cases being referred elsewhere) [14]. These findings highlight that three to five years of diagnostic delay is common globally, with variation by locale and physician awareness. Early manifestations (e.g., isolated oral ulcers) may not immediately fulfil BD criteria, and patients often accrue additional symptoms over months or years before meeting the diagnosis [3]. This prolonged latency is a recognized concern, as delayed diagnosis can postpone treatment and worsen outcomes [13].

Pathophysiology

Recent human studies have advanced our understanding of BD’s complex pathophysiology, implicating dysregulated immunity, vascular injury, and genetic predispositions.

Immune Dysregulation

Mounting evidence indicates BD is driven by an exuberant innate and adaptive immune response. Patients show hyperactive neutrophils and monocytes (with exaggerated pro-inflammatory cytokine release) alongside a skewed Th1/Th17 cell response [15]. Elevated levels of cytokines such as IL-1β, IL-6, TNF-α, IL-17, and others have been consistently observed in active BD [16]. A recent meta-analysis confirmed that BD patients have significantly higher IL-1β, IL-6, and TNF-α compared to healthy controls [16]. There is also evidence of checkpoint pathway dysfunction: the inhibitory PD-1/PD-L1 mechanism (which normally restrains T-cell activation) may be impaired in BD, potentially permitting unchecked Th1/Th17 activity [14]. This dysregulated immune environment underlies the recurrent inflammation seen in BD’s mucocutaneous lesions and organ involvement.

Vascular Inflammation

Behçet’s is a systemic vasculitis that uniquely affects vessels of all sizes, including veins. New findings emphasize the role of neutrophil-endothelial interactions in BD’s vasculopathy. For instance, the integrin α9β1, a molecule aiding neutrophil adhesion to vascular endothelium, is significantly elevated in active BD patients and correlates with inflammatory markers (e.g., high white blood cell and platelet counts) [14]. This suggests neutrophil-driven endothelial injury is central to BD. Indeed, imaging studies have noted increased vein wall thickness in BD patients, reflecting chronic vasculitis of the veins [14]. Such vascular inflammation explains BD’s propensity for thrombosis and aneurysm formation in various organs (e.g., deep vein thromboses, pulmonary artery aneurysms), as inflamed vessel walls contribute to clotting and structural damage. Notably, the disease’s classic lesions (like retinal vasculitis or superficial thrombophlebitis) result directly from this immune-mediated vascular damage.

Genetic Contributions

Genetic predisposition plays a prominent role in BD pathogenesis. The strongest association remains with HLA-B51, an allele enriched along the ancient Silk Road, which confers increased risk for BD [15]. Beyond HLA-B51, recent genomic studies have identified new susceptibility loci linked to disease phenotype. Notably, variations in the MICA gene region (closely tied to HLA-B) and in SLCO4A1 have been associated with a higher risk of BD ocular involvement, whereas polymorphisms in DDX60L are linked to neurological involvement [14,17]. In one 2023 analysis, a single-nucleotide polymorphism (SNP) near SLCO4A1 showed an odds ratio (OR) of ~0.4 for protecting against eye disease, while a variant in DDX60L carried an OR >4 for predisposition to central nervous system (CNS) disease [17]. These findings illustrate that genetic factors influence not only BD susceptibility but also its organ-specific manifestations. Additional studies have highlighted polymorphisms in innate immunity genes (e.g., IL-1 family, IL-10, MEFV) in BD, reinforcing that a genetically primed immune system (especially in populations with those risk alleles) responds abnormally to environmental triggers [15].

Pathophysiological mechanisms and QoL impact

There is a growing recognition that BD’s immunopathology directly translates into reduced QoL for patients. Recent patient-centered research explicitly links the disease’s physical mechanisms to psychosocial outcomes.

Inflammation and QoL

Active disease driven by cytokine and immune dysregulation correlates with worse QoL outcomes. Patients with more severe or active BD (high disease activity scores, reflecting ongoing inflammation) report significantly lower physical and mental health scores on QoL assessments [18]. In a 2023 study, BD sufferers had markedly lower Short Form-36 Health Survey (SF-36) QoL scores than healthy controls across domains like physical functioning, pain, general health, vitality, and mental health [18]. Importantly, higher disease activity was strongly associated with poorer QoL. The study found that patients’ overall physical and mental health scores had a significant inverse correlation with BD activity indices and inflammatory symptoms [18]. This means that the more the immune system is active and causing symptoms, the more patients’ daily functioning and well-being are impaired.

Organ Damage and Specific Symptoms

The manifestations of BD’s pathology (many of which stem from vascular inflammation and autoimmunity) have clear impacts on QoL. Painful mucocutaneous lesions (like oral and genital ulcers), eye involvement, neurological involvement, and arthritis all impose physical limitations and emotional stress. Data confirm that patients with major organ involvement suffer worse QoL. For example, the presence of genital ulcers, ocular lesions, or CNS involvement has been shown to significantly diminish QoL in BD [18]. One cross-sectional study demonstrated that those specific complications were linked to lower SF-36 scores, indicating these pathophysiological manifestations (which can cause pain, vision loss, or neurological deficits) directly lead to poorer QoL [18]. Likewise, patients with sustained arthritis and skin lesions tend to have higher disease activity and report worse QoL than those without such features [19]. In essence, the same immune-mediated damage that causes organ and tissue injury in BD is what drives difficulties in daily activities, work, and social relationships.

Contemporary research reinforces that BD is characterized by long diagnostic delays worldwide, dysregulated immunity (Th1/Th17 hyperactivity, cytokine storms) and vasculitis as key drivers of pathology, and genetic factors that modulate disease expression. These biological mechanisms are not merely laboratory findings - they manifest as painful ulcers, ocular inflammation, vascular occlusions, and other symptoms that significantly undermine patients’ QoL [18]. Recognizing these links is crucial for clinicians to both expedite diagnosis and target the inflammatory pathways in BD, ultimately aiming to improve patient outcomes and daily functioning.

Epidemiology and disease course

BD is recognized worldwide, but its prevalence exhibits significant geographical variation [10]. Turkey (now Türkiye) reports the highest prevalence, with estimates ranging from 80 to 420 cases per 100,000 individuals, depending on the region. In contrast, prevalence rates in other countries along the ancient Silk Road, such as Iran and Japan, are lower, with estimates of 17-68 and seven to 14 cases per 100,000, respectively. In Western countries, the prevalence is considerably lower; for instance, the United States reports estimates ranging from 0.33 to 5.2 cases per 100,000 [20], while Wales (in the UK) has an estimated prevalence of 11.1 per 100,000 [21]. The prevalence of BD varies significantly between and within countries, reflecting a complex interplay of genetic predispositions, such as the higher frequency of HLA-B51 in certain populations [22], environmental influences, and methodological differences in study designs [23,24]. Variability in case ascertainment and diagnostic thresholds further contributes to these disparities. Moreover, clinical manifestations may differ markedly across ethnic, racial, and national groups, complicating disease characterization [10,25].

Clinically, BD follows a waxing and waning course characterized by alternating periods of remission and relapse [26]. During remission, patients often experience near-normal functional capacity and life expectancy, especially when symptoms are effectively controlled through long-term immunosuppressive treatment; however, relapses may lead to severe organ-specific complications [25,27]. Multi-systemic involvement is typical, with the skin, mucosae, and ocular structures being the most affected [28]. Vascular system involvement - such as venous thrombosis, arterial aneurysms, and arterial occlusions - remains the leading cause of morbidity and mortality in BD [29].

Neuro-BD, characterized by neurological involvement, has emerged as one of the leading causes of morbidity and mortality among patients with BD. Neuro-BD occurs in approximately 5%-15% of BD patients and typically manifests with severe complications such as meningoencephalitis, cranial nerve palsies, or cerebrovascular events, significantly impacting patient outcomes and QoL [30,31]. Given its severity, early recognition and aggressive management of neurological symptoms are critical for improving prognosis.

Granulomatous vasculitis, affecting multiple organs, is a key pathogenic mechanism in BD and a research priority due to its significant impact on QoL [29]. Particularly concerning is the impact of BD on renal function. Once azotemia develops in BD patients, progression to dialysis-dependent renal failure typically occurs within a decade if untreated [32].

BD manifestations and disease activity diminish over time. As the disease progresses, the effectiveness of conventional pharmacologic therapies tends to diminish [33]. This underscores the need for early, aggressive, and sustained interventions targeting inflammatory pathways [27]. Given the chronic, relapsing nature of BD and its potential to cause significant physical disability and organ damage, the impact on patients' QoL is profound and multifaceted. Understanding and systematically evaluating QoL in BD is therefore essential for delivering holistic, patient-centered care, beyond solely controlling disease activity [34].

BD is characterized by an unpredictable, relapsing-remitting course that significantly impacts patients' QoL. The variability and uncertainty associated with disease flares can lead to heightened anxiety and hinder the development of consistent coping mechanisms. Patients may adopt various coping strategies; however, certain approaches, like confrontive coping and escape-avoidance, have been linked to increased levels of depression and anxiety, thereby negatively affecting QoL [34,35].

Several factors have been identified as predictors of poor prognosis in BD. Male gender, early age of onset, and major organ involvement-particularly ocular, vascular, and neurological manifestations-are associated with more severe disease courses and poorer outcomes [36]. Additionally, prolonged diagnostic delays can exacerbate disease severity and further diminish QoL. Recognizing these risk factors is crucial for implementing targeted interventions aimed at improving coping capacities, enhancing disease management, and ultimately optimizing QoL outcomes for BD patients [18,37].

Concept and importance of QoL

The concept of QoL has gained notable attention over the past decade, especially regarding chronic diseases [38,39]. It is widely recognized that a chronic condition can significantly shift a person's priorities and influence daily activities related to QoL, as well as challenge their self-perception and identity [40,41]. Chronic illness typically diminishes QoL by impacting emotional well-being, physical and social functioning, and overall life satisfaction [34,39]. As a result, many healthcare professionals now view the assessment of life satisfaction in individuals with complex chronic diseases as crucial [38]. Moreover, such evaluations are generally regarded as reliable indicators for appraising healthcare services [42]. The scientific literature supports that monitoring life satisfaction is integral to delivering appropriate care for those with chronic conditions [42,43].

Health-related QoL in chronic illness

In the context of health care, the term health-related QoL (HR-QoL) is used to understand changes in circumstances that have the potential to lead to or arise from chronic disease; for example, changes in functional ability, psychological or cognitive responses, and/or social function [44]. Although models of HR-QoL differ, they generally assess multiple dimensions, including impairments, functional disabilities, capacity to perform activities, and satisfaction with life [45].

Like all complex subjective measures, HR-QoL comprises a number of dimensions or components. Few reliable and valid HR-QoL measures comprehensively assess all of these components. It is generally held, however, that a full appreciation of chronic disease and the consequent impairment of function on an individual's QoL cannot be made from any single-dimensional perspective [46]. Most evidence on QoL in chronic diseases stems from studies on prevalent public health conditions [45,46]. About BD, epidemiological studies and research dealing with QoL have concentrated on ocular morbidity, aphthous eruption, and joint manifestations [47,48].

Assessment of QoL in BD

Assessing QoL in patients with BD is fundamental to understanding the broader impact of the disease beyond physical symptoms [49]. HR-QoL reflects an individual’s perception of their health burden and encompasses physical, mental, social, emotional, and spiritual dimensions, collectively influencing life satisfaction and perceived happiness [50,51]. In chronic illnesses like BD, improvements in clinical markers alone may be insufficient if QoL remains impaired [52,53]. A statistically significant clinical intervention that does not significantly enhance QoL may be considered insufficiently effective [52]. Consequently, comprehensive evaluation of HR-QoL has become an essential component of both research and clinical practice, as the most meaningful therapeutic outcomes improve both clinical symptoms and HR-QoL [34,52].

Identifying patients with suboptimal QoL scores enables targeted interventions to enhance well-being [34,53]. Regular monitoring of QoL is critical, as changes may signal new disease activity or complications [54]. Importantly, QoL assessments remain the only tools that directly evaluate BD’s impact on patients’ lives, guiding personalized management strategies and predicting future disease burden [34,54,55].

*Approaches to QoL* *Assessment*

QoL in BD can be evaluated through quantitative surveys, structured interviews, or qualitative assessments of patient experiences. The choice of method depends on the research aims, patient population, and desired clinical or research outcomes [56].

*Generic Versus Disease-Specific QoL* *Instruments*

Two main types of QoL assessment instruments are utilized: generic tools and disease-specific tools. Generic instruments, such as SF-36, allow cross-disease comparisons and are widely used in chronic disease research [56-58]. They assess broad domains such as physical functioning, mental health, and social role participation. However, they may fail to capture disease-specific symptoms critical to BD patients, such as oral or genital ulcerations, which are often underrepresented in general health measures [50,58,59]. A summary of the key generic and disease-specific QoL instruments, including their domains, strengths, and limitations, is presented in Table 1.

**Table 1:** Summary of key QoL instruments used in Behçet disease QoL: Quality of Life

Instrument	Domains	Strengths	Limitations
Short Form-36 Health Survey (SF-36)	Physical functioning, role limitations (physical/emotional), bodily pain, general health, vitality, social functioning, mental health.	Allows cross-disease comparisons, widely validated, broadly assesses overall health.	Less sensitive to disease-specific symptoms in BD (e.g., mucosal lesions, ocular manifestations).
Behçet Disease Quality of Life (BDQoL)	Mucosal involvement, pain severity, psychological impact, social stigma, daily functioning.	Highly sensitive to BD-specific impairments, responsive to clinical changes, correlates closely with disease activity.	Limited ability for cross-disease comparisons, less validated in diverse populations.
World Health Organization Quality of Life - Brief (WHOQOL-BREF)	Physical health, psychological health, social relationships, environment.	Broad applicability, validated internationally, comprehensive biopsychosocial coverage.	Generic measure may miss nuances specific to BD symptomatology.

As highlighted in Table 1, disease-specific tools, such as the Behçet's Disease Quality of Life instrument (BDQoL), were developed to address these limitations. BDQoL captures unique burdens experienced by BD patients, including mucosal involvement, pain severity, and social stigma, and demonstrates greater sensitivity to symptom fluctuations over time [34,55,59]. Studies have shown that BDQoL scores correlate closely with disease activity and can guide individualized management strategies [34,53,54].

*Validity, Reliability, and Clinical Utility of QoL* *Instruments*

QoL instruments undergo rigorous processes of reliability and validity testing to ensure their applicability across different populations and clinical settings [59]. Longitudinal tracking of QoL scores in BD patients is clinically valuable, helping to tailor interventions to address the most debilitating symptoms and prioritize improvements in daily functioning and emotional well-being [54,55].

Routine use of both generic and disease-specific QoL assessments in clinical follow-up can enhance patient-centered care, enabling timely therapeutic adjustments and improving long-term outcomes for individuals living with BD. While generic tools help reduce heterogeneity and allow comparisons across different conditions, they often lack the sensitivity needed to capture BD-specific concerns [58]. Disease-specific instruments, on the other hand, are better suited to detect short-term clinical changes, whereas generic measures offer a broader assessment of overall HR-QoL [50,59]. Combining both types of tools provides a more comprehensive evaluation. Building on this understanding, it is crucial to further explore the clinical and psychosocial factors that shape the patient’s experience.

Factors influencing QoL in BD

Physical Symptoms and Disease Burden

Mucocutaneous and ocular involvement represent the most common manifestations of BD, which typically follow a relapsing-remitting course characterized by significant variability in both symptom frequency and intensity. In some patients, multiple organ systems are affected simultaneously during disease flares, while in others, cutaneous manifestations may initially predominate before systemic features emerge. Once established, the disease produces recurrent symptoms that can fluctuate within the same individual over time, with variation observed in the frequency of oral ulcers and erythema nodosum. The severity of clinical presentations can similarly vary between episodes, with the same patient experiencing mild symptoms during one flare and unexpectedly severe manifestations during another. Notably, physical involvement often results in substantial morbidity, contributing significantly to the overall disease burden and impairing QoL [60,61].

Oral ulcers are a common and often debilitating manifestation of BD, causing significant pain and impairing patients' ability to engage socially and interact with their environment. Although their occurrence may seem random and not always proportional to disease severity, their presence contributes substantially to the overall symptom burden, particularly during active disease phases when symptoms intensify. Patients often report fluctuating severity of oral lesions during disease flares. Several factors have been identified as aggravating conditions, including mechanical trauma from tooth brushing and exposure to acidic foods, both of which can exacerbate ulceration and increase patient discomfort [60,62].

Genital ulcers in BD are highly painful and often severely disabling, with most affected individuals reporting that flares involving genital lesions are associated with greater symptom intensity compared to other manifestations. Symptom presence can vary both between different patients and within the same individual across different examinations, with fluctuations often observed between episodes of genital and ocular involvement [63].

Although ocular manifestations are a recognized feature of BD, their reported prevalence tends to be lower across clinical series, possibly because more urgent symptoms affecting other organ systems draw earlier clinical attention; additionally, an increase in neurological complications has been noted in such cases [62,63].

Psychological Disturbances and Mental Health

BD is often associated with chronic pain, disability, and visible symptoms that lead to significant mental health challenges and social stigma [64,65]. Nearly half of BD patients experiencing chronic pain also have a concurrent psychological disorder, highlighting the intertwined nature of physical and psychological suffering [64,66]. Pain relief can significantly influence psychological well-being. Studies indicate that over 50% of BD patients experience depression, with severity correlating with disease activity [67]. Anxiety affects 51%-60% of patients; approximately one-quarter meet criteria for anxiety disorders based on standardized assessments such as the State and Trait Anxiety Inventory, with women reporting slightly higher anxiety levels [67]. Nearly 20% of BD patients use antidepressants or anxiolytics, reflecting the high burden of mood disorders [67].

Negative emotions and cognitive dysfunctions are common in BD, impairing active perception, decision-making, planning, and goal-directed behaviors [68,69]. Functional limitations, including reduced daily activity performance and impaired maintenance of social roles, are frequently reported [68]. The progressive loss of self-identity and autonomy exacerbates psychological distress [69]. Given the bidirectional relationship between physical and psychological suffering, an integrated multidisciplinary approach is essential for optimal patient care [66].

Visible Symptoms and Psychosocial Impact

A study by Senusi et al. [70] demonstrated that facial aphthous ulcers significantly impair psychological health, leading to heightened anxiety, depression, and social withdrawal. The study linked ulcer severity with psychosocial dysfunction and reduced work performance using the Oral Ulcer Severity Score (OUSS) and the Work and Social Adjustment Scale (WSAS). The visible nature of ulcers fosters stigma, fueling feelings of embarrassment, shame, and maladaptive coping behaviors such as substance abuse [70].

Psychiatric Manifestations and Suicide Risk

Beyond depression and anxiety, psychological disturbances in BD include behavioral dysregulation and post-traumatic stress disorder [71]. Notably, suicidal ideation and attempts are significantly more prevalent among BD patients than in the general population [72]. Even patients without formal psychiatric diagnoses often report unhappiness and emotional withdrawal, reflecting maladaptation to the chronic disease burden [73]. Rare but documented psychotic symptoms (e.g., paranoid schizophrenia-like presentations) further complicate the psychiatric profile in BD [71].

Despite their high prevalence, mental health issues in BD are frequently underrecognized in clinical practice. This represents a significant gap, potentially resulting from several barriers, including insufficient clinician awareness, stigma surrounding psychiatric illness, limited access to specialized psychiatric care, and fragmented healthcare delivery. To address these gaps, systematic psychological assessments should become routine in BD management, utilizing validated screening tools such as the Hospital Anxiety and Depression Scale (HADS) or the Beck Depression Inventory (BDI-II) to facilitate early detection and intervention [72,74]. Additionally, healthcare professionals should receive targeted education on the psychiatric aspects of BD, increasing their comfort and competence in managing these manifestations.

Integrating mental health professionals within multidisciplinary teams can also significantly improve care by reducing fragmentation, enabling comprehensive patient assessments, and providing coordinated therapeutic interventions. Furthermore, patient-focused strategies such as psychoeducational programs and cognitive behavioural therapy (CBT) should be incorporated into standard care. These interventions have demonstrated efficacy in improving coping mechanisms, enhancing treatment adherence, and reducing symptoms of depression and anxiety among BD patients [66,75]. Addressing these clinical practice gaps through structured multidisciplinary approaches will not only mitigate psychiatric morbidity but also substantially enhance overall patients’ QoL.

Socioeconomic and Functional Impact

QoL in BD is shaped not only by biological factors but also by profound social and economic determinants [76]. BD progressively deteriorates QoL across personal, occupational, and social domains [48]. Patients commonly report declines in personal and physical fulfilment, perceived health, self-image, time management, sleep quality, and independent living capacity [68,76]. The burden on daily functional activities and disruption of personal customs and roles intensifies as the disease progresses [68].

Employment and Economic Hardship

Functional limitations, particularly due to chronic pain and fatigue, significantly hinder patients' ability to perform daily tasks and sustain employment [68,77]. Pain is frequently cited as a medical basis for work disability, further reinforcing psychological distress and financial insecurity [78].

BD imposes substantial socioeconomic burdens, notably affecting employment rates and economic stability. Studies indicate that approximately 40%-60% of patients with BD experience significant disruptions in their occupational status, with unemployment or underemployment rates markedly higher than the general population. For instance, a recent multicenter study demonstrated that nearly half of BD patients reported work disability or early retirement due to disease severity, particularly in patients with severe organ involvement such as neurological or ocular manifestations [48,77]. Furthermore, economic analyses reveal considerable healthcare expenditures associated with BD management, stemming largely from frequent medical visits, hospitalizations, and specialized treatments, exacerbating the financial hardship experienced by affected individuals and their families [47].

Notably, gender differences in socioeconomic impact have been documented. Women with BD frequently report higher levels of economic vulnerability, partly due to increased susceptibility to psychiatric comorbidities, social stigma, and caregiving responsibilities, leading to greater work absenteeism and lower overall employment rates compared to men [76,79]. Addressing these disparities requires tailored, gender-sensitive interventions to support women in sustaining employment and managing economic stresses.

Given these socioeconomic challenges, targeted interventions including workplace accommodations, vocational rehabilitation programs, and comprehensive disability support systems are crucial. Employers should be encouraged to implement flexible working hours, teleworking options, and physical workspace adjustments to accommodate BD patients’ fluctuating disease activity. Additionally, early vocational rehabilitation and return-to-work programs specifically designed for individuals with chronic inflammatory conditions like BD have shown promise in improving employment retention and economic productivity [78].

Patient advocacy organizations also play a pivotal role in addressing socioeconomic impacts. By raising public awareness, lobbying for improved disability legislation, and providing direct patient support through counselling, legal assistance, and financial advice, these organizations significantly enhance patients' capacity to navigate employment challenges and economic hardships associated with BD [34].

Economic hardship, resulting from job loss, healthcare expenses, and reduced income, acts as a major secondary burden. As patients survive longer due to advances in care, maintaining economic productivity during recurrent disease flares becomes increasingly challenging [77,78].

Addressing Inequities

Patients with greater disability face compounded social exclusion and limited vocational opportunities. Comprehensive management should incorporate early vocational rehabilitation, social reintegration support, and financial counselling alongside medical therapy [77]. Addressing socioeconomic disparities is essential to improving QoL outcomes and reducing secondary psychological morbidity in BD.

Management implications

Given the profound interplay between physical symptoms, mental health, and social factors in BD, optimal management must comprehensively address the totality of the patient experience. Beyond physical symptom control, BD imposes significant psychological and social burdens, necessitating an integrated, multidisciplinary approach. Effective care must simultaneously target the physical, emotional, cognitive, and social dimensions of the disease [66,80].

Pharmacological therapies, including immunosuppressive and anti-inflammatory agents, remain foundational in mitigating disease activity and preventing organ damage [6,27]. However, pharmacologic interventions alone are insufficient to fully restore patients’ QoL, particularly when chronic pain, disability, psychiatric comorbidities, and social stigma persist [65,66]. Early psychological assessment and regular mental health monitoring should therefore be incorporated into routine clinical practice, enabling the timely identification of depression, anxiety, and cognitive dysfunctions that are prevalent among BD patients [72,74]. Moreover, psychoeducational programs have demonstrated value in enhancing coping skills, improving treatment adherence, and fostering psychological resilience in individuals with BD [75].

Encouraging participation in multidisciplinary rehabilitation initiatives, including psychological therapy, pain management programs, and social support interventions, is recommended to comprehensively address the disease burden [70,75]. For example, a psychoeducational program based on the Roy adaptation model significantly improved psychological adaptation and QoL metrics among BD patients [75]. Another successful intervention integrated psychological counselling with pharmacotherapy and pain management, resulting in measurable reductions in depression and anxiety symptoms and enhanced QoL scores over a 6-month follow-up period [66].

Finally, disease-specific QoL assessments should be systematically utilized alongside clinical evaluations to guide personalized treatment plans and to monitor progress over time [34]. By adopting an integrated model of care, clinicians can more effectively mitigate the physical and psychological impacts of BD, improve overall patient satisfaction, and enhance long-term health outcomes.

## Conclusions

BD profoundly impairs patients' QoL through a complex interplay of physical, psychological, and social burdens. Effective management must extend beyond controlling clinical symptoms to delivering comprehensive, multidisciplinary care tailored to individual needs. Incorporating routine QoL assessments, early psychological interventions, and integrated disease management strategies can significantly improve patient long-term outcomes. Future research should prioritize standardizing QoL measurement tools and fostering interdisciplinary collaboration to advance patient-centered care for individuals living with BD globally.
